# Data on metagenomic profiles of activated sludge from a full-scale wastewater treatment plant

**DOI:** 10.1016/j.dib.2017.10.048

**Published:** 2017-10-21

**Authors:** Jianhua Guo, Bing-Jie Ni, Xiaoyu Han, Xueming Chen, Philip Bond, Yongzhen Peng, Zhiguo Yuan

**Affiliations:** a Advanced Water Management Centre (AWMC), The University of Queensland, St Lucia, Brisbane, QLD 4072, Australia; b Key Laboratory of Beijing for Water Quality Science and Water Environmental Recovery Engineering, Engineering Research Center of Beijing, Beijing University of Technology, Beijing 100124, PR China; c Beijing Drainage Group Co., Ltd, Beijing 100022, PR China

## Abstract

The data in this article mainly present the sequences of activated sludge from a full-scale municipal wastewater treatment plant (WWTP) carrying out simultaneous nitrogen and phosphorous removal in Beijing, China. Data include the operational conditions and performance, dominant microbes and taxonomic analysis in this WWTP, and function annotation results based on SEED, Clusters of Orthologous Groups (COG), and Kyoto Encyclopedia of Genes and Genomes (KEGG) databases. Sequencing data were generated by using Illumina HiSeq. 2000 platform according to the recommendations of the manufacturer. The sequencing data have been deposited in MG-RAST server (project ID: mgm4735473.3). For more information, see “Unraveling microbial structure and diversity of activated sludge in a full-scale simultaneous nitrogen and phosphorus removal plant using metagenomic sequencing” by Guo et al. (2017) [1].

**Specifications Table**

**Table:** 

Subject area	*Biology*
More specific subject area	*Biological wastewater treatment*
Type of data	*Table, graph and metagenomic sequences*
*How data was acquired*	*DNA sequencing using Illumina HiSeq. 2000 platform*
Data format	*Raw and filtered*
Experimental factors	*DNA extracted from activated sludge*
Experimental features	*Activated sludge was taken from an aeration tank of a full-scale WWTP in Beijing (China). Metagenomic sequencing was performed using Illumina HiSeq. 2000 platform according to the recommendations of the manufacturer.*
Data source location	*Beijing, China*
Data accessibility	*Data about community structure and function annotation are available with this article. The sequencing data have been deposited in MG-RAST (project ID: mgm4735473.3)*[2].

**Value of the data**

• Data will be useful for investigating microbial community structure in wastewater treatment plants carrying out simultaneous nitrogen and phosphorus removal.
• Data can be used to predict possible nitrogen conversation pathways in biological nitrogen removal systems from wastewater.
• Sequencing data can be used to identify core microbes by comparing to similar data sets generated for simultaneous nitrogen and phosphorus removal plants with different treatment processes.
• Accessibility of metagenomic sequence data allows researchers to perform new analyses with their own research purposes.

## 1 Data

**Fig. 1:**
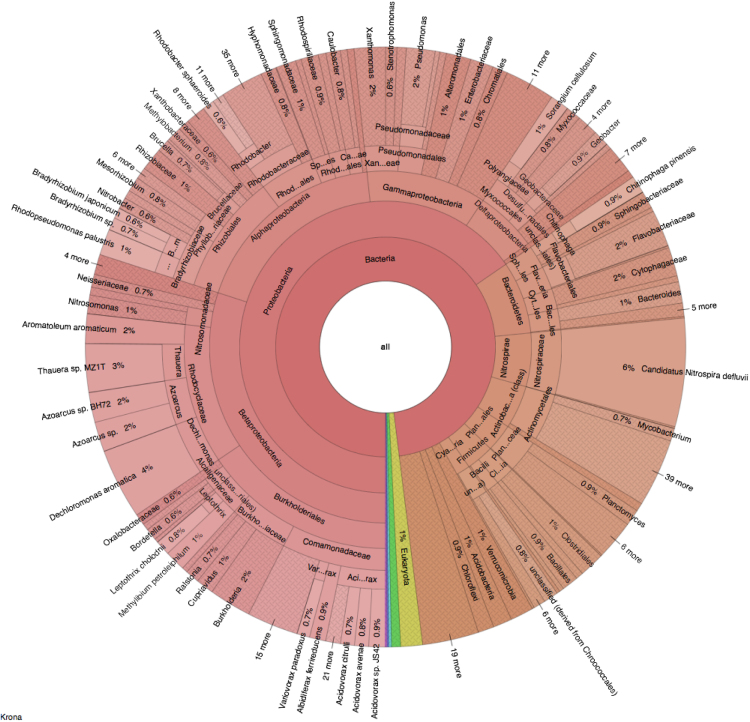
The Krona chart of the full taxonomy.

**Fig. 2:**
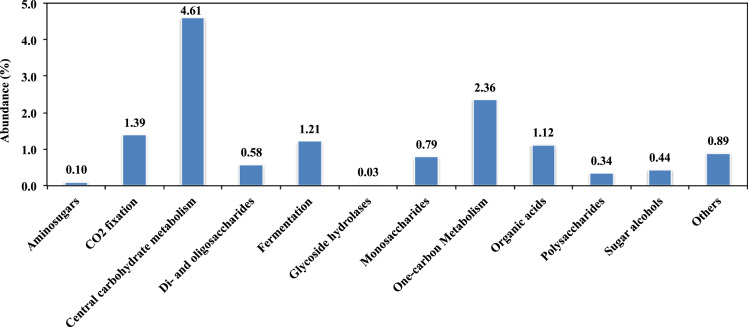
Abundances of major Level 2 subsystems in the sample derived from Level 1 subsystem of carbohydrate based on SEED subsystems (The E-value cutoff of 10^−5^ and minimum alignment length of 17 amino acids was used as the annotation parameters).

**Fig. 3:**
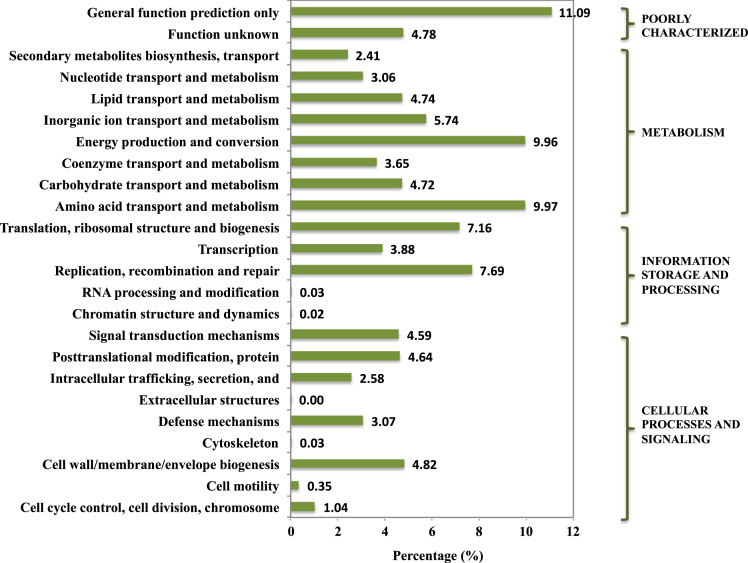
Potential function of genes detected in the activated sludge metagenome based on COG annotation. COG subcategories are listed on the left, and the corresponding major categories are listed on the right.

**Fig. 4:**
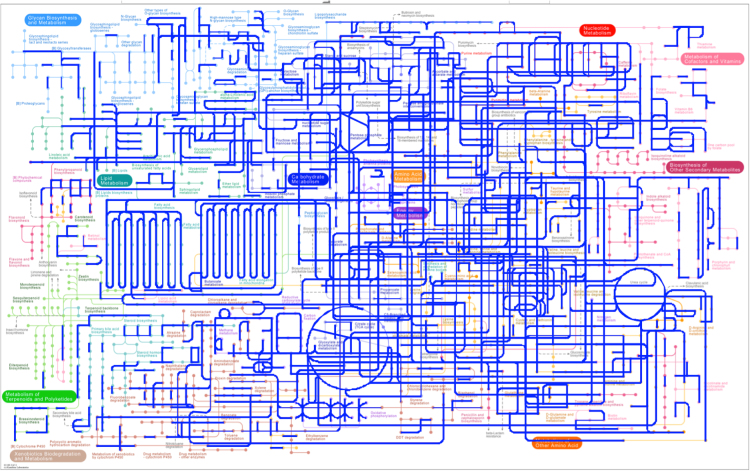
KEGG mapper for the activated sludge. The highlighted line in blue represents the existing pathways in the sample.

**Fig. 5:**
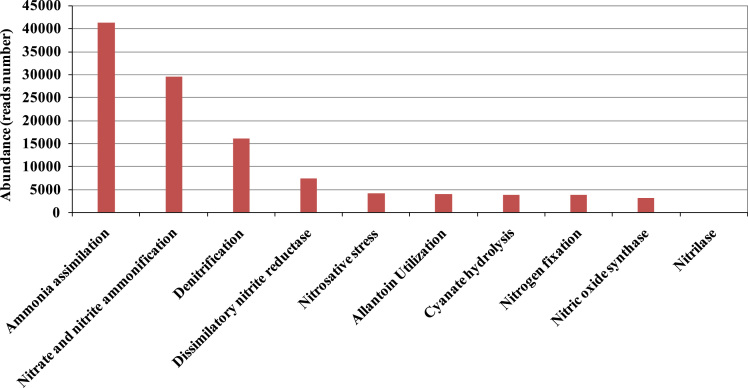
Abundance of nitrogen metabolism sequences from the metagenome based on classification into Level 2 SEED subsystems.

Data on microbial community and functional profiles within activated sludge from a full-scale municipal wastewater treatment plant (WWTP) carrying out simultaneous nitrogen and phosphorous removal (SNPR) are presented [1]. Data include the operational conditions and performance of this WWTP (Table 1), dominant microbes and taxonomic analysis (Table 2 and Fig. 1), and function annotations based on SEED, Clusters of Orthologous Groups (COG), and Kyoto Encyclopedia of Genes and Genomes (KEGG) databases (Fig. 2, Fig. 3, Fig. 4, Fig. 5).

**Table 1:** Operational conditions and pollutant removal performance of the full-scale WWTP (The data are collected from 6 months prior to the sampling).

Unit	T (^o^C)	DO (mg/L)	MLSS (mg/L)	Influent					Effluent						
				COD	BOD_5_	SS	NH_4_^+^-N	TP	COD	BOD_5_	SS	NH_4_^+^-N	NO_2_^-^-N	NO_3_^-^-N	TP
				(mg/L)	(mg/L)	(mg/L)	(mg/L)	(mg/L)	(mg/L)	(mg/L)	(mg/L)	(mg/L)	(mg/L)	(mg/L)	(mg/L)
Range	13.2–24.5	0.3–7.5	3340–5135	167–870	58.6–435.0	90–970	27.4–49.3	2.8–9.0	31.2–46.5	2.1–9.0	8–19	0.2–12.8	0–4.4	12.6–23.3	0.2–1.6
Average	17.6	5.2	4246	432	188.6	320	39.6	5.9	39.2	5.1	12	2.3	0.1	15.8	0.7
STD	3.4	1.5	432	130	63.4	185	4.7	1.1	3.3	1.7	1.9	2.1	0.6	2.0	0.3

T: temperature; MLSS: mixed liquor suspended solid; SS: suspended solids; TP: total phosphorus.

**Table 2:** Abundances of dominant class in the activated sludge sample (the taxonomic classification was preformed by search the contigs against the NCBI NT database using SOAP2 (v2.21, with the default settings).

Phylum	Class	Abundance percentage (%)
*Proteobacteria*	*Betaproteobacteria*	46.19
	*Gammaproteobacteria*	11.14
	*Alphaproteobacteria*	8.19
	*Deltaproteobacteria*	1.51
	*Epsilonproteobacteria*	0.07
*Nitrospirae*	*Nitrospira*	15.4
*Bacteroidetes*	*Flavobacteriia*	3.00
	*Sphingobacteriia*	3.07
	*Cytophagia*	1.44
	*Bacteroidia*	0.32
	*Ignavibacteria*	0.11
*Actinobacteria*	*Actinobacteria*	1.53
	*Gemmatimonadetes*	0.39
	*Acidobacteriia*	0.15
	*Solibacteres*	0.14
*Firmicutes*	*Clostridia*	0.34
	*Bacilli*	0.21
	*Negativicutes*	0.02
*Euryarchaeota*	*Methanomicrobia*	0.07
	*Halobacteria*	0.03
	*Thermoplasmata*	0.03

## 2 Experimental design, materials and methods

### 2.1 Sampling of activated sludge

A 50 mL sample of activated sludge was taken using a plastic dipper from an aeration tank of a full-scale WWTP in Beijing (China). This WWTP treats a mean influent flow of 1×10^6^ m^3^/day. The preliminary wastewater treatment consists of bar screens, aerated grit chambers and primary sedimentation. The plant has an Anaerobic-Anoxic-Oxic (A^2^O) configuration, in which nitrification, denitrification and biological phosphorous removal are simultaneously achieved. The hydraulic retention time is around 6–8 h and the solids retention time is 10–15 days. The excess sludge from the biological treatment settles down in the secondary clarifiers and enters the sludge treatment together. The sludge treatment consists of thickening tanks, anaerobic mesophilic digestion and dewatering.

### 2.2 DNA extraction

Briefly, 2 mL sample was centrifuged at 4000 rpm for 5 min at 4 °C and the sludge pellet was collected. DNA extraction was performed using the FastDNA SPIN Kit for Soil (QBIOgene, Carlsbad, CA, USA) according to the kit manufacturer's instructions. DNA integrity was estimated through gel electrophoresis (1% agarose) and DNA concentrations were measured by using a Qubit Fluorometer (Thermo, USA).

### 2.3 DNA library construction and sequencing

The metagenomic sequencing was performed using Illumina HiSeq. 2000 platform. For library construction, the extracted DNA sample was processed according to the Paired-end Genomic DNA Sample Prep Kit protocol (Illumina) for generating 2×100 bp paired-ends reads. Briefly, DNA fragmentation was performed using the Covaris S2 Ultrasonicator. Then, the DNA fragments were subjected to end-repair, A-tailing, and adapter ligation. After DNA size-selection, PCR amplification and amplicon purification a ~170 bp DNA fragment library was constructed for further sequencing. The base-calling pipeline (version Illumina Pipeline-0.3) was used to generate sequences. In this study, 4.5 Gb reads were generated for the metagenomic dataset. Quality filtering was performed as described previously [3] by removing raw reads that: contained more than 3 ambiguous nucleotides, were shorter than 35 bp, had more than 15 bp overlap with adapter sequences, included more than 36 nucleotides with quality value lower than 20, or were potential duplicated reads due to amplification artifacts. After quality filtering, a total of above 4.0 Gb high-quality DNA reads were used to assemble them into contigs using SOAPdenovo assembler (v 1.05, set as -p 8 -F -M 3 -D 1 -L 90 -u) [4]. The detailed pipeline for bioinformatic analyses can be found in our study [1].
