# Supplementary material for: Data on metagenomic profiles of activated sludge from a full-scale wastewater treatment plant
Source: Data Brief. 2017 Oct 21;15:833–9. doi: 10.1016/j.dib.2017.10.048 (PMC5676080; doi:10.1016/j.dib.2017.10.048)

Conflict of Interest Form

We wish to confirm that there are no known conflicts of interest associated with this publication.

We confirm that the manuscript has been read and approved by all named authors and that there are no other persons who satisfied the criteria for authorship but are not listed. We further confirm that the order of authors listed in the manuscript has been approved by all of us.

Yours,

Jianhua Guo, Dr.

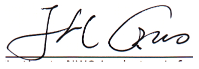

Supplement: Supplementary file 1 — Supplementary material [file mmc1.pdf]
